# A Comprehensive Analysis of Predictors of Marginal Ulcers After Roux-en-Y Gastric Bypass: A Cohort Review of 2106 Patients

**DOI:** 10.3390/medicina62050838

**Published:** 2026-04-28

**Authors:** Tala Abedalqader, Alberto Migliorini, Leonardo Garcia Cerecedo, Nour El Ghazal, Joseph Klim, Tony Boutros, Simon J. Laplante, Omar M. Ghanem

**Affiliations:** Department of Surgery, Mayo Clinic, Rochester, MN 55905, USAelghazal.nour@mayo.edu (N.E.G.);

**Keywords:** marginal ulcer, Roux-en-Y gastric bypass, predictors of marginal ulcer, vagal transection, aspirin, proton pump inhibitors

## Abstract

*Background and Objectives:* Roux-en-Y gastric bypass (RYGB) is one of the most commonly performed metabolic and bariatric surgeries worldwide. Marginal ulcers (MU) are a common complication following RYGB, yet their pathophysiology and the contributing risk factors to their development are not fully understood. *Materials and Methods:* This retrospective cohort study examined patients who underwent RYGB between January 2008 and December 2023, with 1 to 5 years of follow-up. Data collected included patient- and procedure-related risk factors, as well as postoperative MU events. Statistical analysis methods included the independent samples *t*-tests, multivariate regression, and Cox regression analyses. *Results:* Our final cohort included 2106 patients and was predominantly female (80.5%), with a mean age of 47.8 ± 12.1 years and body mass index (BMI) of 45.5 ± 7.5 kg/m^2^. MU occurred in 241 (11.4%) patients, with a mean time to occurrence of 4.5 ± 0.02 years. History of smoking (HR = 1.87, *p* < 0.001) and gastroesophageal reflux disease (HR = 2.36, *p* < 0.001) significantly increased hazard for MU, while proton pump inhibitor use (HR = 0.18, *p* < 0.001) was associated with reduced hazard. Aspirin exposure, regardless of dose and chronicity, did not impact MU. *Conclusions:* Our findings highlight the importance of preoperative assessment and counseling in patients planning to undergo RYGB. Patient-related factors should guide postoperative monitoring and prophylaxis of MU, as this remains a debated topic amongst experts.

## 1. Introduction

Obesity is a chronic disease characterized by a multifactorial etiology and a high prevalence rate in high- and medium-income countries. These two features, together with the pivotal role in the development of other severe conditions, make obesity a global concern [1]. Of the available options for its management, metabolic and bariatric surgery (MBS) stands as the most effective long-term treatment for severe obesity. Currently, laparoscopic Roux-en-Y gastric bypass (RYGB) comprises one of the most commonly performed MBS in the United States and globally, accounting for around 20–25% of all MBS [2].

Despite its significant impact on weight loss and resolution of obesity-related medical conditions, postoperative complications following RYGB are not uncommon in the early or late postoperative period and can result in significant morbidity [3]. Gastrointestinal ulcers, also termed “marginal ulcers” (MU), constitute one of the more frequent late complications [3,4]. They typically develop at the gastrojejunal anastomosis, along the staple line, or involving the surrounding mucosa, especially of the jejunal limb. The pathophysiology of MU is complex, and some risk factors have been identified, including smoking, diabetes mellitus, alcohol consumption, and Helicobacter pylori infection [4].

However, despite the abundance of data on the risk factors that contribute to MU, these studies are not without limitations, such as small sample sizes or short follow-up periods [5,6,7,8,9,10,11,12]. While some studies utilizing the Metabolic and Bariatric Surgery Accreditation Quality Improvement Project (MBSAQIP) database utilized substantial sample sizes, their focus on the early postoperative period leads to missing critical context for the more common late MU occurrence [5,6]. Additionally, most studies lack inclusion or stratification of relevant risk factors. For instance, aspirin is commonly grouped with other non-steroidal anti-inflammatory drugs (NSAIDs) in the literature, and its dose and duration of use are rarely quantified [8,13,14]. Technical factors, such as vagal preservation and intraoperative details, are also often not included in MU risk analysis. Thus, the aim of this study was to provide an encompassing analysis of the impact of both patient-related and procedure-related risk factors on the development of MU following RYGB in a large patient cohort over a long follow-up period. To bridge the gap regarding medication use, data on aspirin dose and chronicity were additionally included and analyzed for their influence on MU occurrence.

## 2. Materials and Methods

### 2.1. Study Design and Patient Selection

Following exemption by the Institutional Board Review (approval date 26 August 2025), a retrospective cohort study of patients aged ≥18 years who underwent primary RYGB at a specialized tertiary center between January 2008 and December 2023 was conducted. All included patients met the criteria for bariatric surgery, including preoperative body mass index (BMI) of ≥30 kg/m^2^, obesity-associated medical conditions, prior weight loss attempts, comprehensive evaluation, and informed consent. Patients who underwent RYGB as a revisional or conversion procedure, had less than one year of postoperative follow-up, or had missing data were excluded from the study.

### 2.2. Data Collection

Data collection was performed through chart review and an institutional data extraction system, using standardized query. The variables collected included baseline data (age, sex, preoperative body weight and BMI, preoperative medications, medical history, and surgical history), intraoperative characteristics (operative time, lysis of adhesions (LOA), and surgical technique), and postoperative variables (medications and complications). Information on postoperative outcomes was tracked and collected using electronic medical records. Patients were followed up at six timepoints (6, 12, 24, 36, 48, and 60 months postoperatively) for at least 1 and up to 5 years; this cap ensured consistency in long-term comparisons given the inclusion of patients from 2008 to 2023.

The primary outcome of interest was the development of MU. Diagnosis was made based on endoscopically confirmed mucosal ulceration at or near the gastrojejunal anastomosis, as documented in endoscopic reports. Endoscopic evaluation was not performed routinely; rather, it was undertaken in patients presenting with symptoms suggestive of MU or when clinically indicated for suspected complications such as stricture or stenosis. Patients were stratified into those who developed MU (MU group) and those who did not (no MU group). Time to occurrence of MU and Clavien–Dindo (CD) classification, applied retrospectively based on the treatment used in the management of MU, were recorded for each patient.

### 2.3. Exposure Definitions

Preoperative risk factors of interest were considered present given clear documentation in the electronic medical record (EMR). History of smoking was defined as reported use of cigarettes within at least one year of operation. Diabetes mellitus (DM) was diagnosed based on the presence of laboratory abnormalities (hemoglobin A1c > 6.5) and the use of anti-diabetic medications, including insulin and non-insulin medications. The presence of gastroesophageal reflux disease (GERD) was defined based on formal diagnosis in the EMR and use of anti-reflux medications. Use of immunosuppressive therapy indicated for transplantation or autoimmune diseases at the time of surgery was noted.

Aspirin exposure included preoperative or postoperative use for >7 days. Low-dose aspirin was defined as 81 mg per day, while high-dose aspirin was defined as any prescription or frequency higher than low-dose. Chronic aspirin exposure included use for >365 days. Oral proton pump inhibitor (PPI) prescriptions (e.g., omeprazole, pantoprazole, lansoprazole, etc.) lasting ≥14 days postoperatively were considered a protective risk factor. Prescriptions lasting <14 days were excluded. If multiple prescriptions for the same medication were noted, the longer one was selected. Open prescriptions with no definitive start or end date and prescriptions starting after MU diagnosis were not counted.

To extract data on surgical approach, operative notes were reviewed to determine whether patients had VS-RYGB (vagal-sparing RYGB) or NVS-RYGB (non-vagal-sparing RYGB) [15]. Additionally, operative time and presence of LOA were recorded from operative notes.

### 2.4. Surgical Technique

Regarding the surgical technique, a 20–30 mL gastric pouch was tailored with multiple staples after the omental split. Then, a Roux limb measuring 100 to 150 cm and a 75 cm biliopancreatic limb were created. The anastomosis was either hand-sewn around a 34F bougie or stapled with an Endo GIA 25 mm stapler (Medtronic, 710 Medtronic Parkway, Minneapolis, MN 55432-5604, USA). An endoscopic leak test and closure of both mesenteric defects were then performed [3].

### 2.5. Study Endpoints

The primary endpoint of this study was to identify predictors associated with the development of MU. An additional secondary endpoint was to examine the influence of aspirin use variability on MU in patient subgroups based on (1) aspirin dose (low-dose vs. high-dose) and (2) chronicity.

### 2.6. Statistical Analysis

Normality of data distribution was assessed using the Kolmogorov–Smirnov test. Data were reported as means and standard deviations (SD) for normally distributed continuous variables or as medians and interquartile ranges (IQR) in variables not normally distributed. Categorical variables were reported as proportions and frequencies (%). Chi-square tests were used to compare rates of categorical variables between those who developed MU versus those who did not, and student *t*-tests were used to compare continuous variables. A *p*-value of <0.05 was set for statistical significance. Multivariate regression and Cox analysis with Kaplan–Meier curves were performed to identify predictors and temporal associations of MU. Statistical analysis was performed using IBM SPSS Statistics for Windows, Version 28.0 (IBM Corp., Armonk, NY, USA).

## 3. Results

### 3.1. Baseline Characteristics

A total of 2106 patients who underwent RYGB during the study period were included in the final cohort. Most of the cohort was female (80.5%), with a mean age of 47.8 ± 12.1 years and a preoperative BMI of 45.5 ± 7.5 kg/m^2^ at the time of the procedure. Diabetes mellitus was present in 29.8% of patients. History of smoking and immunosuppressive therapy was noted in 9.0% and 3.0% of the total cohort, respectively. Table 1 outlines the overall baseline characteristics of all patients.

Aspirin use was noted in 530 (25.2%) patients, either preoperatively or postoperatively, with a median duration of use of 4.1 (0.5, 6.0) years, while 1140 (54.1%) patients had PPI use for a median of 1.0 (0.4, 1.4) year. The majority of patients (n = 1479, 70.2%) underwent NVS-RYGB, while the remaining 554 (26.3%) underwent VS-RYGB. Mean operative time was 162.7 ± 52.9 min, and LOA was performed in 12.5% of patients.

### 3.2. Association Between Marginal Ulcers and Selected Risk Factors

Postoperatively, patients were followed up for a median of 5.0 (3.0–5.0) years, with a retention rate of 71.8% by the end of the follow-up period, reflecting the proportion of patients present at 5 years out of those eligible for follow-up based on their RYGB procedure date. Of the 2106 patients, 241 (11.4%) developed MU, with a mean time to occurrence of 4.5 ± 0.02 years from RYGB. Although a few baseline differences were seen between patients who were and were not retained at the end of the study period, rates of MU development did not differ between the groups (11.6% in patients lost to follow-up vs. 10.2% in patients retained, *p* = 0.388) (Supplementary Table S1). Most cases of MU (n = 182, 75.5%) were classified as CD II, requiring only medical therapy, while the remaining 28 (11.6%) and 31 (12.9%) were CD IIIA and CDIIIB or higher, requiring endoscopic or surgical intervention, respectively.

Univariate comparison of patient- and procedure-related factors showed a significantly higher rate of smoking history (14.9% vs. 8.2%, *p* = 0.001) and preoperative GERD (50.2% vs. 42.5%, *p* = 0.022) in patients who developed MU compared to those who did not. On the other hand, aspirin and PPI use were lower in patients with MU (17.4% and 25.3%, respectively) than patients with no MU (26.2% and 57.9%, *p* = 0.003, <0.001, respectively). Additionally, patients who developed MU underwent NVS-RYGB at a higher rate than patients who did not (78.7% vs. 71.9%, *p* = 0.028). All other risk factors were comparable between groups (Table 2).

Due to substantial missing data, pouch size data was not included and not reported in the analysis. Comparison of patients with and without available data (Supplementary Table S1) demonstrated significant differences between groups. Pouch size data were more frequently available in patients with higher MU rates (12.6% vs. 9.5%, *p* = 0.03) and differed significantly by surgical characteristics, reflecting non-systematic data collection. These findings indicate that missingness was not at random, and inclusion of these variables in multivariable models could introduce bias.

**Figure 1:**
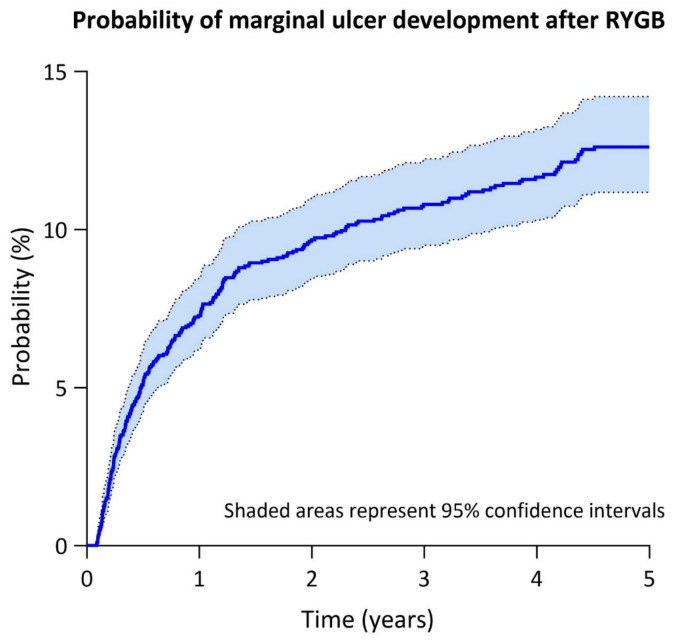
Kaplan–Meier analysis for marginal ulcer development.

Kaplan–Meier analysis (Figure 1) demonstrated an increasing cumulative probability of MU development over the study period, reaching around 9% at 2 years and 11% at 4 years (median not reached). Supplementary Table S2 outlines the number of patients at risk at each study timepoint. The mean time to occurrence, i.e., the period of time in which individuals remained event-free, was 4.5 years. Cox regression analysis (Table 3) was performed to identify factors associated with MU development over time. Multiple factors contributed significantly to the occurrence of MU, including history of smoking (HR = 1.87, *p* < 0.001) and preoperative GERD (HR = 2.36, *p* < 0.001). On the other hand, PPI use (HR = 0.18, *p* < 0.001) was associated with reduced hazard. NVS-RYGB was associated with a HR of 1.27, although this association did not reach statistical significance (*p* = 0.15). Similarly, female sex, preoperative BMI, diabetes mellitus, aspirin use, immunosuppressive therapy, operative time, and LOA were not significant contributors to MU.

Multicollinearity among baseline characteristics and other risk factors in the final Cox regression model was assessed using variance inflation factors (VIF) and collinearity diagnostics. All variables had VIF values close to 1, indicating no evidence of collinearity and confirming model stability. The results of this analysis are provided in Supplementary Table S3.

Patients who developed MU had a median of two risk factors. The rate of MU increased with the number of risk factors present; patients with one, two, three, or four risk factors had MU rate of 9.0%, 13.9%, 15.1%, and 15.4%, respectively. Kaplan–Meier stratification by number of risk factors (Supplementary Figure S1) was also assessed and revealed significant differences in time to MU development between the groups (*p* = 0.0039, log-rank test). Regression analysis showed an increasing HR with the number of risk factors; patients with one, two, three, and four risk factors had an HR of 1.22 (*p* = 0.446), 2.44 (*p* < 0.001), 3.44 (*p* < 0.001) and 3.06 (*p* = 0.043) when compared to no risk factors.

### 3.3. Subgroup Analysis

To discern the influence of aspirin dose and chronicity on MU occurrence, subgroup analysis was performed (Table 4). The rate of MU development was not different when comparing low-dose and high-dose aspirin therapy (8.0% [95% CI: 5.2–10.9] vs. 7.8% [95% CI: 3.6–11.8], *p* = 0.89). Similarly, MU rates were comparable between patients on chronic aspirin therapy and those without chronic use (7.4% [95% CI: 4.0–10.8] vs. 8.3% [95% CI: 5.2–11.5], *p* = 0.69). Aspirin dose and chronicity were not associated with time to MU development. Additionally, to ascertain the potential confounding of PPI on aspirin exposure, a subgroup comparison was done in patients not taking PPI (n = 966). Among those patients, there was no significant difference between patients with aspirin use compared to those without (14.1% vs. 19.8%, *p* = 0.07).

However, differences in patient features were noted on comparison. Age was significantly higher in patients with low-dose (*p* < 0.001) and chronic (*p* = 0.01) aspirin use. Patients with diabetes mellitus were also more commonly on low-dose aspirin (48.2% vs. 29.6% on high-dose aspirin, *p* = 0.00) and chronic therapy (48.7% vs. 37.3% without chronic use, *p* = 0.009). Patients on low-dose aspirin were more frequently chronic aspirin users (46.5%) compared to those on high-dose aspirin (36.7%, *p* = 0.03). Additionally, patients with chronic aspirin use underwent NVS-RYGB more frequently (75.9%) than patients without chronic use (62.8%, *p* = 0.002).

Multivariable logistic regression adjusting for patient- and procedure-related factors demonstrated no significant association between aspirin dose (*p* = 0.29) or chronic use (*p* = 0.05) and MU development.

## 4. Discussion

The present retrospective study provides a comprehensive analysis of the predictors of MU after RYGB in a large cohort of patients over a follow-up period of five years. MU occurred at a rate of 11.4% a median of 4.5 years after RYGB. Multiple factors, including history of smoking and preoperative GERD increased the risk for MU, while PPI use showed a protective effect.

MUs constitute a well-recognized and documented complication following RYGB in the early or, more commonly, late postoperative period. The existing literature has described a mean MU rate of 4.6%, although studies have reported highly variable rates of MU occurrence [4]. A recent systematic review demonstrated an incidence rate of 2.3% to 18.0% across 16 studies [16], with an earlier review reporting up to 25% MU occurrence across 41 studies [17]. This variability has been attributed to differences in sample size, follow-up rate, research year, and definition of MU [4]. In our cohort, MU rate was 11.4%, in congruence with reported data, and occurred a median of 4.5 years following RYGB. Given that endoscopy was performed in a symptom-driven manner, the reported MU incidence likely underestimates the true prevalence due to missed asymptomatic ulcers. Several mechanisms for the occurrence of MU have been suggested, with highly acidic secretions implicated as one of the primary causative factors. Although the bypassing of the antrum in RYGB decreases antral stimulation and subsequent gastrin release, vagal and hormonal stimuli can maintain an acidic environment. High acidity constitutes a particular threat to the jejunal mucosa, which lacks buffering mechanisms against gastric secretions. Clinically, this correlates to MUs more frequently involving the jejunal limb [4,18,19]. Congruently, the presence of preoperative GERD has previously been identified as a risk factor for MU [8,20], a finding that was corroborated by results of our regression analysis. Gilmore et al. attributed this association to an area termed the “acid pocket”, resulting in increased acidity in the proximal stomach, and suggested the use of a smaller gastric pouch to improve this outcome [20].

A technical factor of interest is the role of vagal transection, which has historically been adopted with the goal of reducing the incidence of MU after RYGB [21]. The choice of preservation or transection of the neurovascular bundle remains a widely debated topic and is typically guided by surgeon preference. In a prior cohort study, we observed a higher incidence of MU among patients undergoing NVS-RYGB [15]. In the present analysis, this association (HR = 1.27) was attenuated and did not reach statistical significance after adjustment for patient- and medication-related factors such as PPIs. Furthermore, while a few studies showed a positive correlation between operative time and early MU occurrence [5,6], this association was not found to be significant in our analysis.

Other potential risk factors, including smoking and diabetes mellitus, have previously been linked to the development of MU through their influence on microvascular circulation and subsequent ischemia and inflammation [4,8,22,23,24,25]. No association with DM was found in our study, which could be attributed to differences in glycemic control, duration of disease, or severity of disease, variables that were not collected. History of smoking within one year of operation was identified as a significant contributor to MU occurrence. Although the current practice at our institution requires smoking cessation at least six weeks prior to a planned MBS, this association highlights the chronic and lasting effects of smoking and its profound impact on MU risk, which has been well-documented in the literature. A recent systematic review by Chow et al. demonstrated significant postoperative morbidity and mortality in patients who smoked within 1 year of their MBS [26]. Additionally, Dittrich et al. described a 4.6-fold increase in MU risk in smokers, including light smokers, compared to non-smokers [27]. These findings support the notion of prolonged cessation of smoking in patients with a planned RYGB, beyond the six weeks minimum currently practiced by many institutions [28].

Aspirin use was not found to be a contributing factor to MU development. The role of NSAIDs in ulcer pathophysiology is unclear, with some studies describing an increased risk of MU with their use, resulting from their impact on gastrointestinal mucosal disruption [13,29,30,31], and others finding no association [9,14]. Similarly, several studies have demonstrated an increased incidence of MU with aspirin use [25,30]. Portela et al. described a significant positive association between aspirin use and MU in a systematic review and meta-analysis, but their study was limited by heterogeneity of included studies and the variability in the definitions of low-dose vs. high-dose aspirin, thus resulting in an inability to discern the effects of different dosing on MU development [30]. On the other hand, Di Palma et al. demonstrated aspirin to be protective against MU but suggested their findings might be confounded by the lack of distinction between intermittent and chronic users [8]. Duarte-Chavez revealed a similar relationship and noted a lack of differentiation between doses in their cohort [13]. To fill this gap, the aspirin users in the current cohort were further stratified based on dose and chronicity, with no differences noted in MU rates between subgroups. Low-dose aspirin users in our study were older and had a higher prevalence of diabetes. While neither age nor diabetes was independently associated with MU, the observed lack of association for aspirin may still be influenced by residual confounding. PPI use, which was found to be a significantly protective factor, might additionally represent a confounding factor in our analysis, given that most patients on aspirin were also on PPI therapy. It is possible that longer or more consistent PPI use among aspirin users may partially underestimate the impact of aspirin exposure. This pattern reflects expert recommendations of a longer duration of PPIs prophylaxis in NSAID users to mitigate MU risk [32].

The cumulative effect of risk factors was noted in our analysis, showing a steadily increasing rate of MU the greater the number of risk factors present. A 55.3% increase in MU hazard with each additional risk factor highlights the importance of preoperative evaluation and recognition of potential contributors to MU, as well as close postoperative management and early intervention. That said, it should be noted that the majority of MU cases (87.1%) responded to medical therapy or endoscopic intervention, while 12.9% eventually necessitated surgical intervention, which aligns with published data on revisions for MU [8].

### Strengths and Limitations

The present study provides important and needed context regarding MU, which are a common complication after RYGB. However, some limitations must be acknowledged. The retrospective nature of the study is subject to data collection errors, loss to follow-up, and selection biases. Secondly, some factors affecting MU development, like H. pylori status and alcohol consumption, could not be reliably delineated from medical records. In particular, the lack of alcohol data may have introduced residual confounding, as alcohol use could correlate with other risk factors, including smoking, and thereby influence the observed associations. The exclusion of H. pylori status was primarily related to the long study period, variability in testing methods, incomplete documentation when diagnosis was made at outside institutions, and empiric treatment in the absence of confirmatory testing. Given that testing was not performed systematically and was largely limited to symptomatic patients (13.3% of entire cohort), this may have introduced misclassification, potentially leading to residual confounding and overestimation of associations with other risk factors such as smoking and GERD. Pouch size, which has been demonstrated to be a significant contributor to MU [11], was not included in our analysis due to substantial missing data (~40% of the cohort) and inconsistency in measurement methods, as estimates were often derived from operative notes, while only a small subset of patients had endoscopic measurements. Details on some of the risk factors, such as PPI dose, smoking amount (pack-years), duration, and postoperative relapse were also not included in our data collection. Additionally, patients may be reluctant to disclose discouraged behavior after surgery (smoking, NSAID use), which may potentially underestimate the impact of these factors. Similarly, while data on immunosuppressive therapy was reported, the small sample size (n = 63) may limit its power to detect a true association; thus, the lack of association should be interpreted with caution. Lastly, time-dependent effects of risk factors could not be analyzed and were considered beyond the scope of this study. Despite the aforementioned limits, this study also has strengths, including a comprehensive analysis of MU risk factors in a large sample of patients undergoing RYGB over a long follow-up period and the inclusion of both patient-related and technical factors.

## 5. Conclusions

Based on our results, smoking within one year of RYGB and preoperative GERD has been shown to contribute significantly to the development of MU, while PPI use showed a protective effect. Despite the limitation imposed by the absence of other important risk factors (H. pylori, pouch size and alcohol), our findings highlight the importance of preoperative assessment in patients planning to undergo RYGB, as well as counseling regarding modifiable risk factors. Available evidence demonstrates smoking as far as 1 year before RYGB contributes to MU risk, suggesting that smoking cessation beyond the current minimum requirement of 6 weeks preoperatively may be warranted, although no consensus recommendations exist. Patient-related factors should guide postoperative monitoring and prophylaxis of MU, as this remains a debated topic amongst experts. Further prospective studies including data on pouch size and surgical technique would be vital in fully evaluating contributors to MU after RYGB.

## Figures and Tables

**Table 1:** Baseline preoperative characteristics of the entire cohort.

Variable	Total (n = 2106)
Sex (Female), n (%)	1695 (80.5)
Race (White), n (%)	2018 (95.8)
Ethnicity (Not Hispanic), n (%)	2021 (96.0)
Age at procedure (years), mean ± SD	47.8 ± 12.1
Preoperative BMI (kg/m^2^), mean ± SD	45.5 ± 7.5
Medical history, n (%)	
Diabetes Mellitus	628 (29.8)
Insulin therapy	259 (12.3)
Non-insulin therapy	369 (17.5)
Hypertension	1070 (50.8)
Hyperlipidemia	802 (38.1)
Sleep apnea	1224 (58.1)
History of smoking	189 (9.0)
GERD	913 (43.4)
Immunosuppressive therapy	63 (3.0)

SD: standard deviation, BMI: body mass index, GERD: gastroesophageal reflux disease.

**Table 2:** Univariate comparison of relevant characteristics and risk factors in patients with marginal ulcer vs. patients without marginal ulcer.

Variable	MU (n = 241)	No MU (n = 1865)	*p*-Value †
Sex (Female), n (%)	202 (83.8)	1493 (80.1)	0.165
Age at procedure (years), mean ± SD	48.3 ± 12.1	47.8 ± 12.1	0.59
Preoperative BMI (kg/m^2^), mean ± SD	44.8 ± 7.9	45.6 ± 7.44	0.12
Diabetes Mellitus, n (%)	71 (29.5)	557 (29.9)	0.897
*Insulin therapy*	30 (12.4)	229 (12.3)	0.85
History of smoking, n (%)	36 (14.9)	153 (8.2)	**0.001**
GERD, n (%)	121 (50.2)	792 (42.5)	**0.022**
Immunosuppressive therapy, n (%)	11 (4.6)	52 (2.8)	0.128
Aspirin therapy, n (%)	42 (17.4)	488 (26.2)	**0.003**
PPI therapy, n (%)	61 (25.3)	1079 (57.9)	**<0.001**
NVS-RYGB ^+^, n (%)	185 (78.7)	1291 (71.9)	**0.028**
Operative time (min), mean ± SD	164.6 ± 49.0	162.5 ± 53.4	0.547
Lysis of adhesions, n (%)	32 (13.3)	232 (12.4)	0.711

MU: marginal ulcer, SD: standard deviation, BMI: body mass index, GERD: gastroesophageal reflux disease, PPI: proton pump inhibitor, NVS-RYGB: non-vagal-sparing Roux-en-Y gastric bypass. † Pearson’s chi-squared test for categorical variables, independent samples *t*-test for continuous variables. Bolded *p*-values are statistically significant. ^+^ Missing data for 6 patients with MU and 70 with no MU. Italicized text indicates a subgroup of Diabetes Mellitus.

**Table 3:** Cox regression for the development of marginal ulcer.

Variable	HR ^‡^	LL 95% CI	UL 95% CI	*p*-Value
Female sex	1.13	0.79	1.62	0.50
Preoperative BMI	0.99	0.97	1.01	0.19
History of smoking	1.87	1.31	2.68	**<0.001**
Diabetes mellitus	1.06	0.79	1.42	0.70
GERD	2.36	1.78	3.12	**<0.001**
Immunosuppressive therapy	1.20	0.63	2.28	0.57
Aspirin therapy	0.71	0.50	1.00	0.05
PPI therapy	0.18	0.13	0.25	**<0.001**
NVS-RYGB	1.27	0.92	1.74	0.15
Operative time	1.00	0.99	1.00	0.35
Lysis of adhesions	0.97	0.65	1.45	0.88

HR: hazard ratio, LL: lower limit, UL: upper limit, CI: confidence interval, BMI: body mass index, GERD: gastroesophageal reflux disease, PPI: proton pump inhibitor, NVS-RYGB: non-vagal-sparing Roux-en-Y gastric bypass. ^‡^ Adjusted hazard ratio after controlling for baseline characteristics. Bolded *p*-values are statistically significant.

**Table 4:** Subgroup analysis based on aspirin dose and chronicity.

**Dose**			
Variable	Low-dose aspirin (n = 361)	High-dose aspirin (n = 169)	*p*-value †
MU rate, n (% [95% CI])	29 (8.0 [5.2–10.9])	13 (7.7% [3.6–11.8])	0.89
Time to MU (years), mean ± SD	4.7 ± 0.1	4.7 + 0.1	0.95
Age at procedure (years), mean ± SD	53.2 ± 11.1	49.4 ± 10.7	**<0.001**
Preoperative BMI (kg/m^2^), mean ± SD	45.2 ± 7.8	44.5 ± 6.7	0.32
Female sex, n (%)	276 (76.5)	135 (79.9)	0.38
History of smoking, n (%)	29 (8.0)	19 (11.2)	0.23
Diabetes mellitus, n (%)	174 (48.2)	50 (29.6)	**<0.001**
GERD, n (%)	152 (42.1)	81 (47.9)	0.21
Immunosuppressive therapy, n (%)	9 (2.5)	7 (4.1)	0.30
Chronic aspirin therapy, n (%)	168 (46.5)	62 (36.7)	**0.03**
PPI therapy, n (%)	212 (58.7)	113 (66.9)	0.07
NVS-RYGB, n (%)	237 (69.7)	109 (65.7)	0.36
**Chronicity**			
	No chronic aspirin (n = 300)	Chronic aspirin (n = 230)	*p*-value
MU rate, n (% [95% CI])	25 (8.3 [5.2–11.5])	17 (7.4 [4.0–10.8])	0.69
Time to MU (years), mean ± SD	4.7 ± 0.1	4.7 ± 0.1	0.66
Age at procedure (years), mean ± SD	50.9 ± 11.7	53.4 ± 10.2	**0.01**
Preoperative BMI (kg/m^2^), mean ± SD	44.9 ± 7.6	45.0 ± 7.2	0.85
Female sex, n (%)	240 (80.0)	171 (74.3)	0.12
History of smoking, n (%)	27 (9.0)	21 (9.1)	0.96
Diabetes mellitus, n (%)	112 (37.3)	112 (48.7)	**0.009**
GERD, n (%)	134 (44.7)	99 (43.0)	0.71
Immunosuppressive therapy, n (%)	9 (3.0)	7 (3.0)	0.98
High-dose aspirin therapy, n (%)	107 (35.7)	62 (27.0)	**0.03**
PPI therapy, n (%)	191 (63.7)	134 (58.3)	0.21
NVS-RYGB, n (%)	182 (62.8)	164 (75.9)	**0.002**

MU: marginal ulcer, SD: standard deviation, BMI: body mass index, GERD: gastroesophageal reflux disease, PPI: proton pump inhibitor, NVS-RYGB: non-vagal-sparing Roux-en-Y gastric bypass. † Pearson’s chi-squared test for categorical variables, student *t*-test for continuous variables. Bolded *p*-values are statistically significant.

## Data Availability

The datasets generated and analyzed during the current study are not publicly available because of the need to protect patient privacy and confidentiality following the ethical standards and regulatory requirements but are available from the corresponding author upon reasonable request.

## Supplementary Materials

The following supporting information can be downloaded at https://www.mdpi.com/article/10.3390/medicina62050838/s1, Table S1: Comparison of characteristics between patients with and without select data; Table S2: Number of patients at risk at each time-point corresponding to Kaplan Meier curve (Figure 1); Table S3: Variance inflation factors (VIF) and collinearity diagnostics; Figure S1: Kaplan-Meier stratification by number of risk factors.

## Abbreviations

The following abbreviations are used in this manuscript:

MBS	Metabolic and bariatric surgery
RYGB	Roux-en-Y gastric bypass
MU	Marginal ulcer
MBSAQIP	Metabolic and bariatric surgery accreditation quality improvement program
NSAID	Non-steroidal anti-inflammatory drug
BMI	Body mass index
LOA	Lysis of adhesions
CD	Clavien-Dindo
EMR	Electronic medical record
DM	Diabetes mellitus
GERD	Gastroesophageal reflux disease
PPI	Proton pump inhibitor
VS-RYGB	Vagal-sparing Roux-en-Y gastric bypass
NVS-RYGB	Non-vagal-sparing Roux-en-Y gastric bypass
SD	Standard deviation
IQR	Interquartile range
