# Supplementary material for: A Comprehensive Analysis of Predictors of Marginal Ulcers After Roux-en-Y Gastric Bypass: A Cohort Review of 2106 Patients
Source: Medicina (Kaunas). 2026 Apr 28;62(5):838. doi: 10.3390/medicina62050838 (PMC13208551; doi:10.3390/medicina62050838)

**Supplementary Table S1**: Comparison of characteristics between patients with and without select data			
**Variable**	**Pouch size data available**	**No pouch size data**	**p-value**
MU rate, n (%)	12.6	9.5	0.03*
Sex (Female), n (%)	79.9	81.5	0.36
Age at procedure (years), mean ± SD	47.9 ± 12.0	47.8 ± 12.3	0.90
Preoperative BMI (kg/m^2), mean ± SD	45.8 ± 7.8	45.0 ± 6.7	0.009*
Medical history, n (%)			
Diabetes Mellitus	29.4	26.1	0.11
Hypertension	51.6	49.5	0.37
Hyperlipidemia	38.3	37.7	0.82
Sleep apnea	58.7	57.2	0.52
History of smoking			
GERD	44.1	42.0	0.37
Immunosuppressive therapy	3.4	2.3	0.15
Aspirin use	24.0	27.1	0.12
PPI use	53.4	55.3	0.42
NVS-RYGB	95.3	33.0	0.00*
**Variable**	**Retained patients (n = 626)**	**Patients lost to follow-up (n = 1480)**	**p-value**
MU rate, n (%)	11.0	12.5	0.37
Sex (Female), n (%)	80.3	81.0	0.72
Age at procedure (years), mean ± SD	48.3 ± 12.0	46.9 ± 12.4	0.01*
Preoperative BMI (kg/m^2), mean ± SD	45.4 ± 7.4	45.8 ± 7.7	0.22
Medical history, n (%)			
Diabetes Mellitus	29.5	24.9	0.03*
Hypertension	51.8	48.4	0.15
Hyperlipidemia	39.9	33.7	0.008*
Sleep apnea	59.8	54.2	0.02*
History of smoking	26.7	21.6	0.01*
GERD	43.9	42.0	0.44
Immunosuppressive therapy	3.1	2.7	0.68
Aspirin use	26.7	21.6	0.24
NVS-RYGB	74.0	69.7	0.05
MU: marginal ulcer, SD: standard deviation, BMI: body mass index, GERD: gastroesophageal reflux disease, PPI: proton pump inhibitor, NVS-RYGB: non-vagal-sparing Roux-en-Y gastric bypass<br>**Denotes statistical significance*			

**Supplementary Table S2**: Number of patients at risk at each time-point corresponding to Kaplan Meier curve (figure 1)	
**Time**	**Patients at risk (n)**
0 months	**2086**
6 months	**1980**
12 months	**1900**
24 months	**1731**
36 months	**1521**
48 months	**1329**
60 months	**1099**

**Supplementary Table S3:** Variance inflation factors (VIF) and collinearity diagnostics				
**Variable**	**HR / Coefficient**	**95% CI (Lower – Upper)**	**p-value**	**VIF**
Gender (Female)	1.01	0.98 – 1.05	0.54	1.07
Preop BMI	0.999	0.997 – 1.003	0.12	1.04
Diabetes mellitus	1.007	0.976 – 1.038	0.65	1.07
Immunosuppressive therapy	1.024	0.945 – 1.103	0.55	1.01
GERD	1.09	1.06 – 1.12	<0.001	1.22
Aspirin use	0.967	0.935 – 0.998	0.04	1.04
Smoking	1.082	1.035 – 1.129	<0.001	1.01
NVS-RYGB	1.022	0.992 – 1.053	0.15	1.03
Operative time	1.00007	1.000 – 1.000	0.60	1.11
LOA	1.005	0.963 – 1.047	0.83	1.09
PPI use	0.846	0.806 – 0.865	<0.001	1.20

HR: hazards ratio, CI: confidence interval, VIF: variance inflation factor, BMI: body mass index, GERD: gastroesophageal reflux disease, NVS-RYGB: non-vagal-sparing Roux-en-Y gastric bypass, LOA: lysis of adhesions, PPI: proton pump inhibitor

Supplementary Figure S1: Kaplan-Meier stratification by number of risk factors

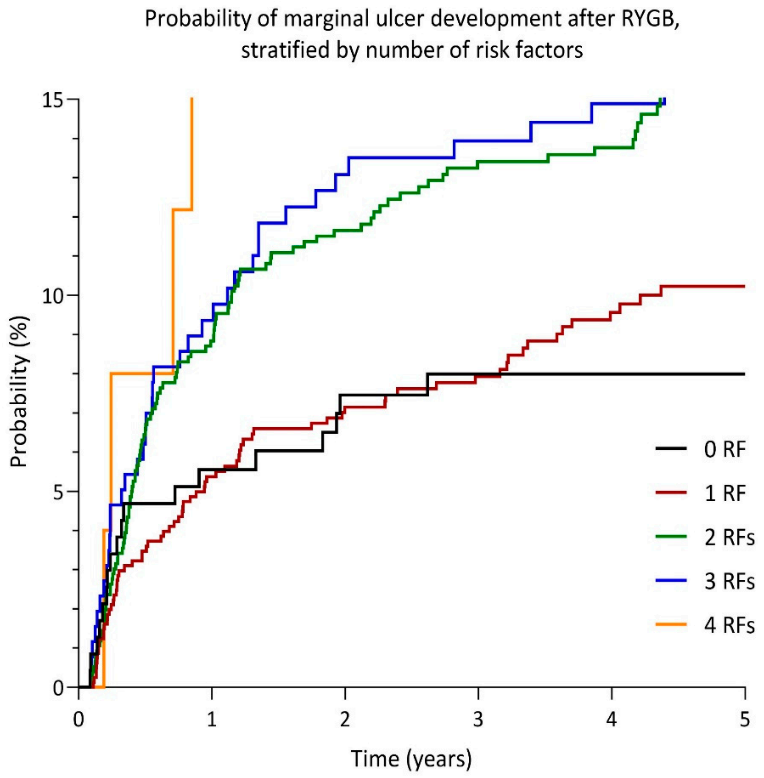

Supplement: Supplementary file 1 [file medicina-62-00838-s001.zip › medicina-4198234-supplementary.pdf]
